# High patient acceptance of immediately sequential bilateral cataract surgery (ISBCS) as part of a one-stop see-and-treat pathway within an innovative NHS cataract unit

**DOI:** 10.1038/s41433-024-03567-3

**Published:** 2025-01-02

**Authors:** Maher Alsusa, Shakeel Ahmad, Zoe Smith, Sam Evans, Elizabeth Wilkinson, Harry Roberts

**Affiliations:** 1 University of Exeter Medical School, Exeter, UK; 2 West of England Eye Unit, Royal Devon University Healthcare NHS Foundation Trust, Exeter, UK

**Keywords:** Surgery, Health services

## Abstract

**Background:**

Constituting ~0.5% of all NHS cataract operations, national provision of immediately sequential bilateral cataract surgery (ISBCS) is limited. Combining offering ISBCS within a novel one-stop see-and-treat (S&T) cataract pathway would offer patients the opportunity for two cataract operations in a single hospital visit. Patient acceptance of ISBCS amongst urban populations has been investigated. However, little is understood about ISBCS acceptance rurally.

**Methods:**

Retrospective observational study at the Nightingale Hospital, Exeter investigating patient acceptance of ISBCS within S&T; following the implementation of a S&T cataract pathway entailing a pre-operative patient-clinician telephone consultation and subsequently scheduled single date of assessment and surgery. Patient acceptance and factors potentially influencing decisions were investigated.

**Results:**

200 patient telephone consultations between 22nd August 2023 and 9th January 2024 were evaluated. 198 (99%) patients referred were suitable for S&T cataract surgery, of whom 109 (54.5%) were deemed eligible for offering ISBCS S&T cataract surgery. Of the eligible participants, 78 (71.56%) favoured ISBCS. No significant differences in age, sex, distance from hospital or refractive data were identified between ISBCS accepting and declining participants.

**Conclusions:**

Our results illustrate a high patient acceptance rate (71.56%) of ISBCS within our population in contrast with published national rates. Offering ISBCS within a S&T model would allow patients to benefit from having both cataracts assessed and treated within a single hospital visit.

## Introduction

Cataract surgery remains the most performed surgical procedure worldwide [1]. Considering the aging British population and ever-growing waiting lists, NHS cost burdens are projected to increase by 50% in the coming decades, further exacerbating the existing disequilibrium between demands and service provision [2, 3]. As such, an exploration into novel approaches within cataract services is warranted. In the UK, bilateral cataracts are typically treated by operating on each eye across two separate hospital visits, also known as delayed sequential bilateral cataract surgery (DSBCS) [4]. Immediate sequential bilateral cataract surgery (ISBCS) entails performing cataract surgery on both eyes within a single theatre session and has gained popularity in recent years with significant research favouring its implementation [5–7].

Benefits of ISBCS to patients include reduced hospital visits, quicker visual rehabilitation, avoidance of anisometropia and reduced waiting times [8]. From a healthcare provider perspective, ISBCS has also been shown to boost theatre productivity and more efficiently manage resources [9, 10]. ISBCS is supported both by the National Institute for Health and Care Excellence (NICE) and the Royal College of Ophthalmologists (RCOphth) [11]. Although the RCOphth has encouraged ISBCS to help tackle backlogs resulting post-pandemic [12], uptake is still limited as suggested by recent National Ophthalmology Database (NOD) data and represents less than 1% of all UK cataract surgery [1, 13]. Acceptance of ISBCS to UK patients has previously been investigated, most recently by Malcolm et al. in a London population which showed patient preference for ISBCS in 55.6% of patients [8].

**Fig. 1:**
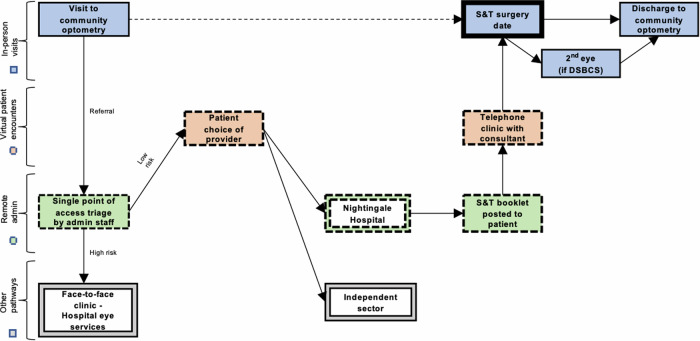
Nightingale Hospital, Exeter—See-and-Treat pathway.

Long waiting lists and clinical pressures are important factors in the evolution of more efficient clinical pathways. In the last year our cataract service has adopted a ‘See-and-Treat’ (S&T) model for cataract referrals pre-screened through a single point of access team by a consultant ophthalmologist as ‘low risk’. Those not triaged into the S&T model are offered a traditional face to face appointment. Briefly, S&T patients are sent an information pack by post or email (See-and-Treat Booklet—Supplementary Material 1), are signposted to the NHS cataract decision tool (Supplementary Material 2—Decision support tool: making a decision about cataracts) and receive a scheduled telephone call by a consultant ophthalmologist. During the phone call the consultant will discuss the patients’ symptoms, their willingness for the S&T pathway, reiterate the material in the information pack, discuss the risks and benefits of routine cataract surgery and discuss preferred refractive outcomes as well as answering any questions from the patients (Fig. 1—Nightingale Hospital, Exeter—See-and-Treat pathway).

To our knowledge, there is no published research investigating patient acceptance of ISBCS outside of a face-to-face consultation. This study aims to explore the preferences of patients offered ISBCS as part of a preliminary phone call prior to expected S&T cataract surgery.

## Methods

### Study design

This was a retrospective observational study investigating patient acceptance of ISBCS within a S&T pathway at the Exeter NHS Nightingale Hospital of the Royal Devon University Healthcare NHS Foundation Trust (RDUH). The methods of this study adhered to the Declaration of Helsinki and approval was given by the RDUH Quality Improvement Department (reference number: 23–977).

### Study procedure

Inclusion criteria for this study included all consecutive patients in the S&T pathway telephone consultation consultant-led clinics over a 6-month period. Phone calls were scheduled for 15-min slots and a consultant would be templated to conduct 12 phone calls per clinic. The content covered in each telephone consultation is detailed in Table 1.

**Table 1:** Structure of Telephone Consultation.

• Confirms patient details and agreement with content in referral letter
• Ensures patient has received, read and understood S&T cataract resources
• Establishes symptoms and willingness for surgery
• Past ophthalmic history
• Establishes consent with S&T model
• Discusses risks and benefits of cataract surgery
• Discusses refractive outcome and documents provisional refractive target
• If suitable for offering ISBCS, discusses patient options of ISBCS or DSBCS, the advantages of each and records patients’ preference.
• If patients’ preference is for S&T ISBCS then discusses that if additional conditions are discovered on the day of surgery, they may not be eligible for surgery on one/both eyes.
• Any additional questions arising from the S&T booklet or from the phone call are answered.

Participants were excluded from the study based on modified criteria originating from RCOphth [12], shown in Table 2, as well as patients with insufficient contralateral cataract (based on contralateral visual acuity ≥6/7.5 and absence of significant symptoms).

**Table 2:** Exclusion criteria for offering ISBCS.

• Insufficient contralateral cataract/unilateral pseudophakia
• Lives alone/lacks sufficient care in home environment
• Presence of ophthalmic conditions increasing risk of postoperative complications or refractive surprise, such as (but not limited to):
○ Corneal oedema (significant Fuchs endothelial dystrophy, previous keratoplasty)
○ Postoperative uveitis (significant uveitis history)
○ Cystoid macular oedema (significant epiretinal membrane/vitreomacular traction/retinal vascular disorders or significant diabetic retinopathy)
○ Refractive surprise (corneal dystrophies or significant keratoconus)
○ Increased risk of endophthalmitis (significant blepharitis, poor personal hygiene/neglect)

Patients expressing a preference for ISBCS were warned that suitability would only be confirmed on the day of surgery through clinical examination and investigations, and if unsuitable then would be offered DSBCS if appropriate. All patients on the S&T pathway were counselled to expect that as the clinical examination is performed on the same day as cataract surgery that the planned outcome may change on the day of assessment. Participants then had a single date of assessment and operation scheduled by the booking team.

### Data collection

Participant data was extracted through accessing electronic patient records (Epic, Epic Systems Corp., Verona WI, USA) retrospectively. Categories of individual variables obtained from patient records include date of birth, sex, date of telephone consultation and postcode. Reasons for ISBCS unsuitability, patient preference of unilateral/DSBCS/ISBCS, provisional target refraction and spherical equivalent of subjective refraction were obtained (from the optometry referral letter).

Participant postcode was collected to calculate patient travel distance to the site of operation. This was calculated by inputting participant postcodes and the hospital postcode into Google Maps (Alphabet Inc., Mountain View CA, USA) through a private browser on a shared trust device. Descriptive statistics were used to calculate eligibility, ineligibility and the patient acceptance rate of ISBCS. Normality of continuous data (age, distance from hospital, spherical equivalents) was assessed using Shapiro-Wilk tests. Parametric and non-parametric data were subsequently assessed with unpaired *t*-tests and Mann-Whitney U tests respectively. Categorical data (sex, preliminary target refraction) were assessed with chi-squared tests. All statistical tests were written and executed in Python (PSF, Wilmington DE, USA) on Visual Studio (Microsoft Corp., Redmond WA, USA). Statistical significance was set at *p* < 0.05.

## Results

200 consecutive pre-operative phone call appointments conducted by a consultant ophthalmologist were evaluated between 22nd August 2023 and 9th January 2024 representing 18 clinics worth of phone calls.

### Patient characteristics

**Fig. 2:**
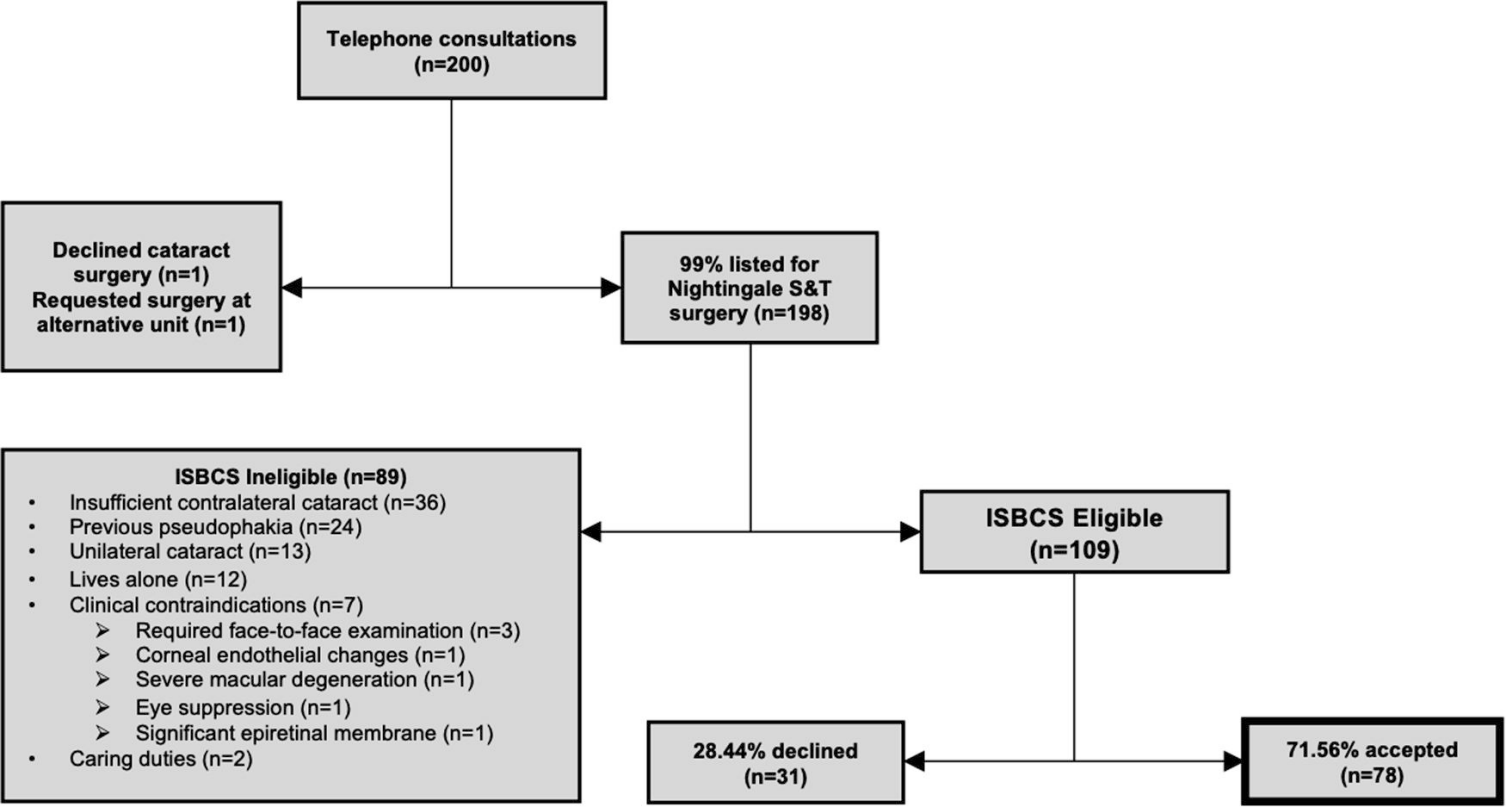
Patient acceptance of S&T ISBCS and factors influencing eligibility.

Male and female participants were 106 (53%) and 94 (47%) in number respectively. The mean age of all participants was 76 (±7.3) years. The average distance required to travel for cataract surgery was 22.4 (±17.6) miles. Of all 200 participants, 109 (54.5%) were eligible for offering ISBCS. 89 (44.5%) participants were ineligible for offering ISBCS. Patients were excluded from offering ISBCS based on the following reasons: likely insufficient contralateral cataract (*n* = 49, 55.1%), referred for second eye cataract surgery (*n* = 24, 27%), living alone or with very dependent spouse with limited social support (*n* = 14, 15.7%), unsuitable for clinical reasons (*n* = 7, 7.9%). Of the patients who were excluded due to insufficient contralateral cataract, three also lived alone and two also had spousal caring duties. The sum of percentages exceeds 100% since some participants met more than one exclusion criterion. Of the 109 patients who were offered choice of unilateral/DSBCS or ISBCS, the acceptance rate of ISBCS was 78/109 (71.56%). The ultimate conversion rate from optometry referrals to our S&T pathway was 99%, with two patients not listed for S&T. Figure 2 offers a complete breakdown of patients’ acceptance rates and factors influencing eligibility.

There were no significant differences in the demographics of those preferring ISBCS against those preferring unilateral/DSBCS. Table 3 illustrates a comparison of both participant groups based on each characteristic.

**Table 3:** Characteristics of ISBCS accepting or declining participants.

Characteristic	ISBCS Accepting	ISBCS Declining	*P*-value
Sex, *n* (%)^a^			0.641
Male	44 (56.4%)	19 (61.3%)	
Female	34 (43.6%)	12 (38.7%)	
Age (Years), mean (±SD)^b^	77 (±7)	77 (±6.3)	0.997
Distance from unit, (miles), mean (±SD)^c^	21.7 (±18.2)	23 (±19.5)	0.822
Arithmetic mean SE (D), (±SD)^c,d^			
Left	−0.49 (±3.51)	−0.43 (±2.96)	0.923
Right	−0.31 (±3.39)	−0.76 (±2.91)	0.371
Absolute mean SE (D), (±SD)^c^			
Left	2.48 (±2.51)	2.13 (±2.07)	0.668
Right	2.56 (±2.22)	2.26 (±1.94)	0.497
Preliminary target refraction, *n* (%)^a^			0.557
Emmetropia	75 (96.2%)	29 (93.5%)	
Myopia	3 (3.8%)	2 (6.5%)	

*ISBCS* immediately sequential bilateral cataract surgery, *SE* spherical equivalent.

Statistical tests used to determine *p* value:

^a^Chi-squared test.

^b^Unpaired t-test.

^c^Mann-Whitney U test.

^d^Spherical equivalent data missing for one participant.

## Discussion

Data from the NOD 2023 describes that the proportion of cataract patients who underwent ISBCS is ~0.5%, a rate that has remained stagnant when compared with previously published figures [1, 13]. Such data suggests that there are significant barriers in place to the adoption of ISBCS in the UK, despite support from bodies such as RCOphth and NICE as well as evidence demonstrating no increased risk, boosted theatre efficiency and patient preference [5, 8, 9, 14].

Our study involved 200 preliminary phone calls between patients referred for S&T cataract surgery at the NHS Nightingale Hospital, Exeter and a consultant ophthalmic surgeon in which 71.56% of eligible patients preferred the opportunity for ISBCS over a unilateral/DSBCS approach. This rate of acceptance exceeds that of 55.6% reported recently by Malcolm et al. in a London population; although there are a number of important differences between the studies [8]. These include an urban (London) rather than a rural (Devon) population, as well as being counselled for surgery in a face to face clinic setting by a variety of clinicians (including non-surgeons) versus a telephone pre-assessment by one consultant surgeon experienced with ISBCS. The age and sex distribution appeared similar across the two studies as well as to NOD 2023 [13]. Regardless of the differences between the studies, both indicate high rates of patient acceptance of ISBCS in stark contrast with ~0.5% of ISBCS performed in the UK. Malcolm et al. reported that of those patients listed for ISBCS, 40% felt they were recommended ISBCS by their clinician. In contrast with our study the surgeon did not recommend either course, but offered patients either option followed by an explanation of the advantages, disadvantages and risks of either approach.

We hypothesised that in our rural county with relatively poor infrastructure, patients with further to travel may express a stronger preference for ISBCS but this was not the case with an average distance to travel of 21.7 (±18.2) miles for accepting participants compared to 23 (±19.5) miles for ISBCS declining participants (*p* = 0.882). Equally, we did not observe a difference in preferences based on age (*p* = 0.997) or sex (*p* = 0.641). Despite evidence suggesting females and older people are more risk averse [15, 16] our results are likely due to the context-dependency of patient decisions. High myopes were expected to favour ISBCS more strongly, however no significant differences in ISBCS acceptance were identified based on spherical equivalents of left (*p* = 0.923) or right (*p* = 0.371) eyes. Similarly, preliminary target refraction did not seem to significantly have an impact on patient acceptance of ISBCS (*p* = 0.557). A relatively equal degree of deviation from emmetropia was identified across both accepting and declining participants, with no significant difference between both left (*p* = 0.668) and right (*p* = 0.497) eyes. Seemingly, degree of pre-operative ametropia did not appear to play into patient decisions regarding ISBCS. This perhaps indicates overarching reasons for opting for ISBCS in our population are related to convenience rather than concerns of intra-operative anisometropia.

An important novelty of our research is the patients expressed their preference as part of a pre-assessment phone call in a S&T pathway. In a more traditional cataract pathway, the discussion would occur following a face to face history, slit lamp examination, biometry and ancillary investigations and cataract counselling. Presumably, this would introduce additional opportunities for the patient’s baseline preference on ISBCS to be influenced, either positively or negatively. Whether a difference in acceptance rates via a telephone consultation compared with clinic appointments is impactful remains to be investigated. S&T pathways occur throughout medicine, most notably for the management of minor injuries within an Emergency Department [17]. However, S&T has demonstrated efficacy in skin cancer excision services – successfully reducing appointments and waiting times with >80% of patients being treated on the first visit [18]. Ultimately, S&T aims to expedite patient care, improve patient convenience without compromising on safety or clinical outcomes. Successful integration of a S&T ISBCS pathway would offer a cataract pathway limited to a single patient hospital attendance. The benefits of this to both patients and a public healthcare system such as the NHS would likely be profound [19].

A large minority (44.5%) of patients were not offered ISBCS based on the aforementioned exclusion criteria. Given the novelty of a S&T pathway offering possible ISBCS via a telephone appointment, we determined to be relatively conservative in our approach but our reasons for not offering ISBCS were clear and consistent. Whilst advisory bodies have encouraged ISBCS when suitable, NICE have advised against ISBCS in patients with high risk of ocular complications [11]. Similarly, RCOphth guidance advises against discussing consent for one-stop ISBCS solely on the day of surgery [12]; our design highlights the benefit and patient acceptability of the consent discussion occurring in advance via a telephone appointment. In this patient cohort of newly referred cataract patients who were triaged into a low risk pathway, incidence of significant comorbidities affecting the surgical plan were sufficiently rare. The remarkably high conversion rate (99%) from referral to S&T pathway listing is evidence of the high-quality referrals from local optometrists, followed by a robust triage system to allocate patients to the S&T pathway safely and efficiently.

### Limitations

Participants included were under the care of a single consultant experienced in ISBCS, perhaps leading to reduced generalisability of our results to other clinicians. Our cohort of cataract patients arose from new referrals from community optometry who were triaged by consultant ophthalmologists into the low risk S&T pathway, hindering the applicability of our results to more clinically complex patients and those with comorbidities or alternative referral routes. Similarly, considering our participants were sourced from a Devon population, our acceptance rates may not be reflected in more demographically varied areas of the country. The alternative logistical design of S&T could impact the applicability of our findings to traditional face-to-face cataract clinic pathways. Patient viewpoints regarding our proposed cataract pathway were not sought as to determine patient rationale for opting in or out of ISBCS. Economic analysis of our novel pathway including assessment of the administrative costs of phone clinics vs face to face clinics was not performed as this was deemed beyond the scope of this study.

## Conclusion

There is a very high acceptance rate of ISBCS in our population. This is a significant finding since it is in contrast with the overall low national rates of ISBCS in the UK. Both ISBCS and S&T convey multiple benefits to patients, whilst also offering cost-saving and efficiency benefits for the healthcare system alongside having a lower carbon footprint. Based on our results we would advocate for wider investigations into acceptance of ISBCS and/or S&T within a UK population.

## Summary

### What was known before

- ISBCS constitutes a significantly small proportion of all NHS cataract operations.
- ISBCS offers multi-level benefits to patients and healthcare providers.
- Strong acceptance of ISBCS has been demonstrated amongst patients of urban populations.

### What this study adds

- Our study demonstrates high patient acceptance rates of ISBCS within a S&T pathway in a rural population.
- S&T pathways are a feasible and effective healthcare model to deliver cataract surgery.
- S&T cataract pathways can facilitate cataract management within a single hospital visit.

## Supplementary information

Supplemental Material 1 - See-and-treat patient booklet, Nightingale Hospital, Exeter

Supplemental Material 2 - Decision support tool: making a decision about cataracts

## Data Availability

Data available upon written request to the corresponding author.
