# Correction: High patient acceptance of immediately sequential bilateral cataract surgery (ISBCS) as part of a one-stop see-and-treat pathway within an innovative NHS cataract unit

**DOI:** 10.1038/s41433-025-03700-w

**Published:** 2025-04-09

**Authors:** Maher Alsusa, Shakeel Ahmad, Zoe Smith, Sam Evans, Elizabeth Wilkinson, Harry Roberts

**Affiliations:** 1 University of Exeter Medical School, Exeter, UK; 2 West of England Eye Unit, Royal Devon University Healthcare NHS Foundation Trust, Exeter, UK; 3 Faculty of Health and Life Sciences, University of Exeter, Exeter, UK

**Keywords:** Surgery, Health services

Correction to: *Eye* 10.1038/s41433-024-03567-3, published online 2 January 2025

An affiliation change for author Harry Roberts to change his affiliation of ‘University of Exeter Medical School, Exeter, UK’ to ‘Faculty of Health and Life Sciences, University of Exeter, Exeter, UK’

Please note the other affiliation of Harry Roberts should remain as it is (West of England Eye Unit, Royal Devon University Healthcare NHS Foundation Trust, Exeter, UK).

