# Supplementary material for: High patient acceptance of immediately sequential bilateral cataract surgery (ISBCS) as part of a one-stop see-and-treat pathway within an innovative NHS cataract unit
Source: Eye (Lond). 2025 Jan 2;39(6):1165–9. doi: 10.1038/s41433-024-03567-3 (PMC11978750; doi:10.1038/s41433-024-03567-3)
Supplement: Supplementary file 1 — Supplemental Material 1 - See-and-treat patient booklet, Nightingale Hospital, Exeter [file 41433_2024_3567_MOESM1_ESM.pdf]

NHS
Royal Devon
University Healthcare
NHS Foundation Trust

Patient Information

CEE See and Treat Cataract Surgery

Reference Number: RDE 24 010 001
(version date: March 2024)

What's included in this leaflet

The see and treat process........ 1
The telephone appointment........ 1
Important things to discuss........ 2
Risks of surgery........ 2
Where we are........ 3
Arriving on the day........ 4
Meeting the surgeon........ 4
During the surgery........ 5
After the surgery........ 5
Eye drops........ 5
Follow-up appointments........ 6
Glasses after surgery........ 6
Showering/swimming........ 7
Driving........ 7
General Guidance........ 7
What is normal for the next few days?........ 8
What is **not** normal after the operation?........ 9
Useful contacts........ 10

This booklet contains important information to help you decide if cataract surgery is right for you. Please read it carefully and in full.

You have been referred for consideration of cataract surgery by your optometrist. In order to provide a streamlined service, we have adopted a “see and treat” approach to cataract surgery. This is a proven and safe model for us to deliver surgery to our patients, however you may be aware of some differences from the normal approach, when you might be seen in clinic at the hospital prior to surgery.

The NHS has a cataract decision making tool which we highly recommend you read before your first appointment by entering the following web address into your device;
www.england.nhs.uk/publication/decision-support-tool-making-a-decision-about-cataracts/
This will tell you whether cataract surgery will be beneficial for you.

The see and treat process

See and treat surgery minimises the number of times you need to come to hospital. You will only need to have a telephone consultation followed by your surgery.

The telephone appointment

After we have received your referral from your optometrist, you will receive an appointment for a telephone call from an experienced member of the team. During this telephone call you will be asked about the problems you are having with your eyesight, and the effect of these problems on your life.

You might want to discuss things such as:

- The problems you are having with your vision
- Whether one or both eyes are affected
- Whether there are any tasks you are no longer able to perform (i.e. driving)

If on the other hand you are generally happy with your vision then please mention this too. If you are not having any problems then surgery is probably not for you!

Important things to discuss

Please tell us if any of the following apply to you:

- You've had any other eye surgery, including laser surgery to correct a glasses prescription
- You wear contact lenses:
    - Soft contact lenses – remove 1 week before surgery
    - Hard contact lenses – remove 4 weeks before surgery
- You take Tamsulosin, Prazosin, Doxazosin or Alfuzosin
- You are allergic to anything

Risks of surgery

During the call we will explain the risks of surgery. It's very important that you are aware of these and ask any questions that concern you. The risks of surgery are:

- Lack of improvement in your eyesight (1 in 50 cases)
- Loss of vision (1 in 100 cases)
- Further surgery (1 in 100 cases)
- Blindness or loss of the eye (1 in 3,000 cases)

Cataract surgery is the most commonly performed and one of the safest operations in the UK. Over 95% of patients undergoing cataract

surgery do not experience any complications. Although the risks are small, it’s very important that you only have surgery if you feel they are worth taking in order to try to improve your symptoms.

After discussing your symptoms over the phone you will both make a decision about having surgery. If you decide to go ahead you will be sent an appointment to attend the Nightingale Hospital for same day assessment and surgery.

Where we are

The Centre of Excellence for Eyes is a department within the Nightingale Hospital Exeter. We are on Osprey Road in Sowton Industrial Estate, just off junction 30 of the M5.

The letter you will receive when your appointment is booked includes more information on where and how to attend for your surgery.

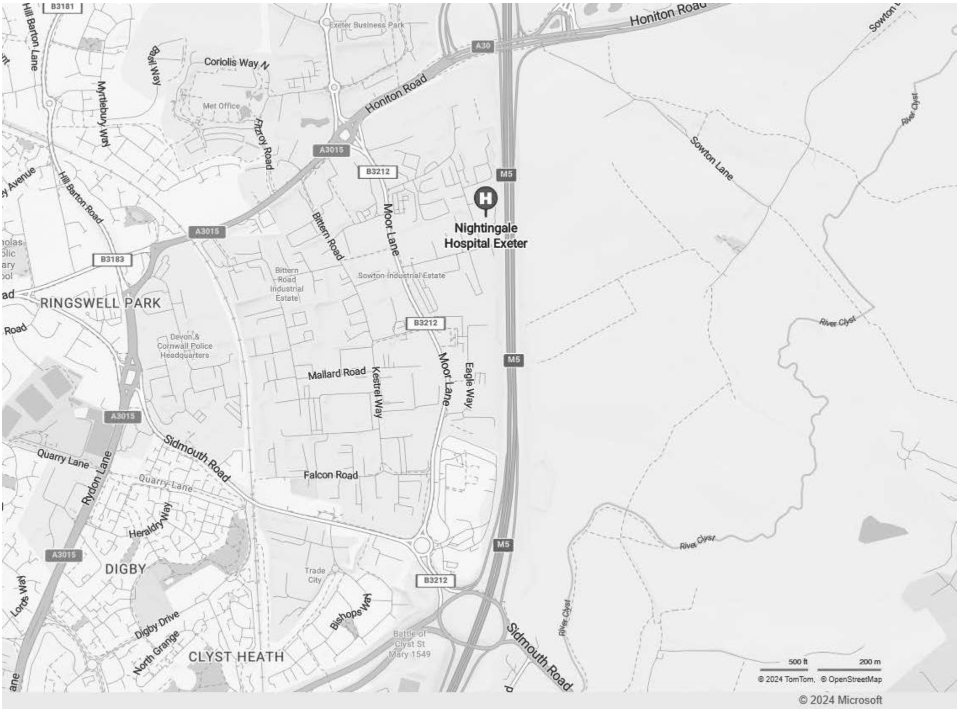

Arriving on the day

You will be greeted by a receptionist at the Surgical Reception where your details will be checked. You will then follow the yellow line to the Centre of Excellence for Eyes (CEE) where you will be met by one of the nursing team, who will confirm your identity and carry out some basic observations such as blood pressure and blood sugar (if you are diabetic). You will have your vision checked and some measurements of your eyes taken (biometry) and some scans taken (OCT). These are to check the health of your eye and to assist in the planning of the surgery.

Meeting the surgeon

Following the scans, a member of the surgical team will introduce themselves to you and examine your eyes in order to confirm that surgery is likely to benefit you, and to look for signs of any other eye disease. This is the same examination that would have been carried out had you have been seen in a traditional clinic. It is possible that your surgeon may find evidence of other eye disease which may impact the decision to operate. They will discuss their findings with you.

If the decision is to continue to surgery - as it usually is - then the risks of surgery will be discussed with you again so that you can sign your form to consent to surgery. It is important that you understand these risks and feel that your eyesight is poor enough that you are happy to take these risks.

In rare cases the surgeon may feel that the cataract is not the cause of your vision problems. If that is the case then they will advise you that surgery is not likely to help, and will suggest the correct route forward - which may be an appointment in another clinic in the eye department or discharge back to your optometrist. This does not happen often, but until your eye has been examined it cannot be ruled out.

During the surgery

The surgery usually takes around ten minutes, and is usually comfortable. You will be brought into our bespoke operating theatre and made comfortable on the operating bed. Your surgeon will clean around your eye and then put a sticky blanket over your face in order to keep the operation sterile. Many people worry about this stage, but in fact it is not unpleasant and your surgeon will make sure you are comfortable before starting the operation. You will be aware of a very bright light and may feel some pressure or slight stinging sensations and water running during surgery.

If you need to cough or move during surgery, simply raise your hand and let the team know.

After removing the sticky blanket at the end of the operation (patients often say this is the most uncomfortable part of the procedure!) you will be taken back to the waiting room for a cup of tea or coffee. You will be able to contact whoever is taking you home and wait in the unit until they arrive.

After the surgery

Eye drops

We suggest you go home and take things easy for the rest of the day.

Wash your hands before applying any eye drops. If you notice a build-up or crusting on or around the lashes then you can bathe the eyelids with salted cooled boiled water and clean cotton wool.

You can start your eye drops on the same day as the surgery. If you normally use any other eye drops then it is usually best to start a new bottle. If you are applying multiple eye drops then please leave 5 minutes between drops.

If you have blepharitis, continue with your lid hygiene but be gentle.

It's common for some eyedrops to burn or sting on instillation. You should continue your drops as prescribed. However, if your discomfort seems to be worsening, please contact the department. Burning/stinging or a gritty feeling may also be a symptom of dry eyes and artificial tears can be used. These are available from any pharmacy.

You should have enough eye drops to last you for a month. You can get a repeat prescription from your GP. Occasionally people are allergic to the drops. If your eye becomes red, waters a lot or the eye lids swell after using the drops, please contact the department for advice.

Follow-up appointments

Once both cataracts have been treated you will be discharged from the hospital. Our specialist surgeons request you have a sight test at 6 weeks with your optician as you may require an updated glasses prescription.

If the surgeon wants to see you again after surgery those arrangements will be confirmed with you before you leave the unit. If you attend the hospital for other eye conditions (e.g. glaucoma) then these appointments should continue uninterrupted. If you have any queries please discuss these with your surgical team.

Glasses after surgery

During cataract surgery it's possible to correct your glasses prescription. It's important to consider the ideal glasses prescription to fit your lifestyle. For example, near focus for sewing, reading or art, or far focus for birdwatching, driving and general activities. It's not possible to be glasses free entirely, and you may need glasses for all activities after surgery.

Some specialist lens types, for example, toric lenses which correct astigmatism, and lenses which increase the depth of focus (EDOF and Multifocal), are not routinely available on the NHS, but the lenses we

use at the Nightingale have a proven track record of safety and are very likely to give you a great result.

You will be able to get a new glasses prescription for between 4-6 weeks after your operation for the second eye. You could otherwise buy an off the shelf pair of reading glasses in the meantime.

Showering/swimming

You may shower after 24 hours but avoid getting water directly in the eye for 7 days. You should avoid going swimming or to a sauna for four weeks after the operation.

Driving

As long as you meet the DVLA eyesight requirements, i.e. to be able to read a number plate at a distance of 20 metres with both eyes open, you should be able to drive 48 hours after your surgery. If you have any concerns about how other eye conditions may affect your driving, please discuss this with the team.

General guidance

- Lying on your side in bed, reading or watching television does not harm the eye
- You cannot strain the eye or wear it out by using it or wearing the wrong prescription glasses
- Try not to rub or knock the eye after surgery but you may gently wipe or dab around the eye
- Be careful not to get anything dirty or dusty in the eye whilst on the drops, such as from DIY or gardening
- You may use eye make-up with care four weeks after the operation
- If you enjoy specific hobbies or activities, please ask for advice on resuming this after your operation

- Avoid anything unnecessarily strenuous, but generally speaking you are extremely unlikely to harm the eye by any sort of physical activity.

What is normal for the next few days?

- Your eye may feel sore or tender
- You may feel as if there is something in the eye like a lash or piece of grit
- You may have a slight headache
- The white of the eye may look red, and this may last for up to 2 weeks
- The eye may be watery and have some mucous in the corner
- You may experience a flickering sensation
- You may find lights very bright
- The pupil of the eye may remain dilated for 1-2 days
- You may be aware that the vision is not balanced
- You may be more aware of floaters

All of these should gradually improve, **nothing should get worse**. If you are at all worried, please contact the department immediately.

What is not normal after the operation?

- Pain in or around the eye or a throbbing ache not eased by simple pain killers such as paracetamol
- If your eye is sticky with a yellow discharge
- If the eye becomes increasingly red
- If the eye is feeling gritty and this is getting worse
- If your eyesight gradually becomes more blurred or there is a loss of vision
- If you cannot tolerate bright light and this is getting worse
- Feeling sick in the first 24 hours after the operation

If you have any of the above problems, **please contact the department immediately on the number below**. Please be prepared to come back for a check-up that day.

Useful contacts

If you are at all concerned after your surgery and wish to speak with a member of the team, please contact the unit's administrative team on the below number between 08:00-16:00 Monday-Friday so that they can direct your call to the appropriate person or service:

01392 908641

If there's no answer on the number above, please leave a message and we will get back to you.

If your query is urgent, or you need advice **outside of normal working hours**, please call **111.**

We are very proud of the See and Treat service that we provide at the Nightingale Hospital as we believe it provides safe, effective and convenient surgery for our patients.

We are always looking for ways to improve the service and would welcome any feedback that you may have. We encourage you to record any comments on a patient feedback card available in the unit.

This information can be offered in other formats on request, including a language other than English and Braille.

RD&E main switchboard: **01392 411611**

For Royal Devon services log on to: https://royaldevon.nhs.uk

Smoking and second-hand smoke causes harm to patients and staff, and is not allowed on any Trust sites.

The Trust cannot accept any responsibility for the accuracy of the information given if the leaflet is not used by Royal Devon staff undertaking procedures at the Royal Devon hospitals.

© Royal Devon University Healthcare NHS Foundation Trust

Designed by Graphics (Print & Design), RD&E (Heavitree)
