# Supplementary material for: High patient acceptance of immediately sequential bilateral cataract surgery (ISBCS) as part of a one-stop see-and-treat pathway within an innovative NHS cataract unit
Source: Eye (Lond). 2025 Jan 2;39(6):1165–9. doi: 10.1038/s41433-024-03567-3 (PMC11978750; doi:10.1038/s41433-024-03567-3)
Supplement: Supplementary file 2 — Supplemental Material 2 - Decision support tool: making a decision about cataracts [file 41433_2024_3567_MOESM2_ESM.pdf]

Making a decision about **Cataracts**

NHS

What is this document?

This document is called a decision aid. It will help you decide between different treatments for cataracts. You should go through it and then talk to your optometrist (optician) or specialist to make a decision together.

1. **Read pages 1 – 5** to help you decide what to do about treatment.
2. **The last 6 pages** give you additional useful information.

This decision aid is for you if your specialist or optician has told you that you have cataracts and **cataracts are causing you problems** that are affecting you doing day-to-day tasks such as reading, driving, watching TV or doing puzzles.

1 What are your options?

Do nothing	Use aids or adaptations	Have cataract surgery

What are cataracts?

Cataracts are a part of the natural aging process where the lens in the eye becomes cloudy. This clouding is called a cataract.

Having some clouding or cataract in the eye is not harmful in itself and does not mean you need to have them removed. Having cataracts does not mean you will go blind.

How did I get cataracts?

The cloudy patches (cataracts) gradually build up in everyone's eyes from about age 40. Over time they can get worse and affect how well we can see. See page 6 for more information.

If cataracts are causing you problems in your day to day life you might want choose a treatment option.

2 What are your options?

What can I do myself?

Cataracts can worsen due to things like poor diet, smoking, UV light from the sun and poorly controlled diabetes. More of what you can do to slow down development of cataracts is on pages 6 and 7. There are no medicines that can slow down cataracts.

Do nothing

You can choose to do nothing about your cataracts. Your cataracts will not harm your eye, but your eyesight might get worse.

Aids and adaptations

These help you make the most of the sight that you have. You can try these before or instead of surgery.

Adaptations include:

- using large print books
- making text larger on screens
- use better lighting.

Aids include:

- prescription glasses
- magnifying lenses
- sunglasses and hats to prevent glare, or block sunlight.

Cataract surgery

It is usually done under local anaesthetic (numb the eye, you are not put to sleep). The surgery itself takes around 30 minutes. Your cloudy lens is removed and is replaced with a clear plastic lens.

Not everyone will be able to see better after surgery, around 80 – 90 out of every 100 people will be able to see better.

You will not be able to drive yourself home and your vision will be blurry for up to 4 weeks. Healing will usually take 4 – 6 weeks.

You can drive, fly, and go back to work as soon as you can see well enough.

3 What's important to you?

It's up to you to decide if or when is the best time to have cataract surgery. There is no 'right time'. Think about whether your cataracts are affecting your day-to-day life and then talk to your optometrist or specialist. Talk to family or friends you know you have had surgery.

For each question **put an ✘ where your answer lies.**

	Yes	No
I can see well through both eyes	☐	☐
I am unaffected by sunlight or glare while driving	☐	☐
I am able to fully enjoy watching TV, reading and doing close-up work	☐	☐
I can easily see the edges of steps	☐	☐
I have several other health conditions that may affect my eye health e.g. diabetes macular degeneration, glaucoma	☐	☐
It is hard for me to take time off work to have surgery, or to cope with the recovery period	☐	☐
I am worried about the possible risks of the surgery (see page 5)	☐	☐

More 'Yes' answers:

for you, doing nothing, or vision aids & adaptations may be good options

More 'No' answers:

for you, cataract surgery may be worth considering

Now read the next page to compare the potential benefits and risks

4 Potential benefits and risks

The numbers here come from research. See page 10 for links to the studies.

Use aids or adaptations

You can see well enough, although you may use aids and adaptations for some tasks.

How many people **out of 100** can read a newspaper **with a vision aid** who couldn't read it without an aid?

55 can

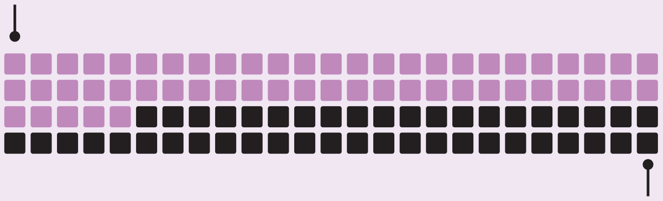

45 can't

Cataract Surgery

You cannot see well enough for tasks with aids and adaptations.

Out of every **100 cataract surgeries:**

around 87 have eyesight **better** than before (sharper and clearer)

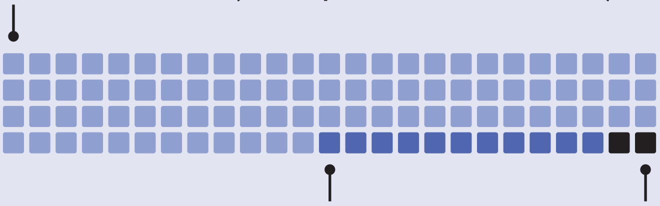

around 11 have eyesight **the same** as before

around 2 have eyesight that is **worse** than before

What do people say about their eyesight afterwards?

Out of every **100 people trying low vision aids and adaptations:**

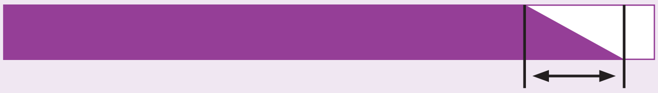

80 95

80 – 95 say that they have a benefit.

Out of every **100 people asked about their vision after cataract surgery:**

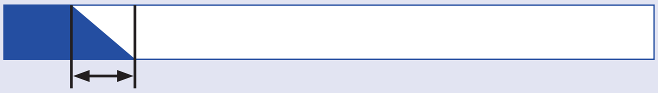

10 20

10 – 20 say they didn't feel benefit from surgery because of problems like glare or shadows.

Your sight might be sharper and clearer than before (able to read the chart), but it might feel worse.

5 Potential benefits and risks

Drawbacks or risks of the treatment options

Use aids or adaptations

There are no risks to trying aids to help you see better but there might be drawbacks.

- You may need to pay for new glasses or specialist visual aids.
- Your vision is likely to continue to get worse.
- There may come a point where glasses or other corrections can't improve your vision enough and it might start affecting your day to day life, for example being able to drive or read.
- Some people find it hard to get used to visual aids.

Cataract Surgery

Surgery is not risk free and results are not guaranteed.

How many people suffer complications because of the surgery?

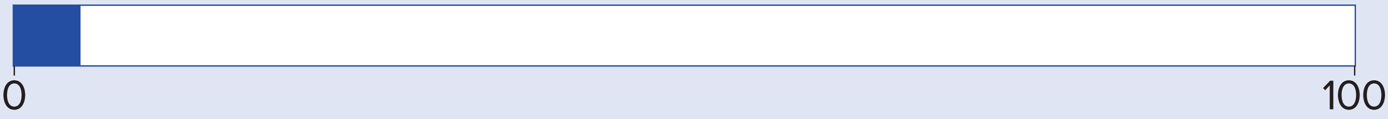

Around 5 in every 100 have some form of complication, such as infection (endophthalmitis: 0.3 out of 100) or detached retina (0.3 out of 100). **These can be treated but vision is often poor after this kind of complication.**

How many people suffer clouding of the new lens after surgery?

Some have clouding behind the new lens (not a new cataract) which can be removed with laser treatment if necessary. This happens to:

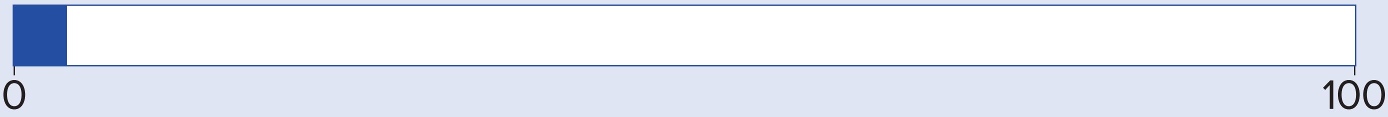

around 4 in every 100 after the **1st year**

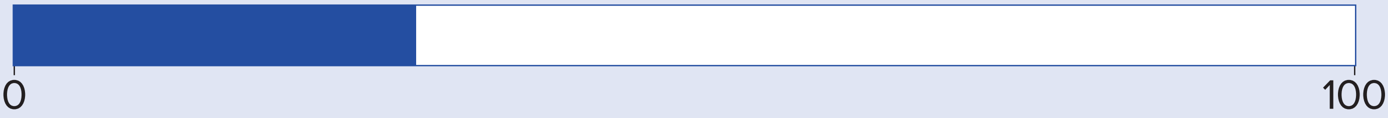

and around 30 in every 100 by the end of **5 years**

6 Cataracts in more detail

Cataracts are very common. **Over 400,000 cataract surgeries are done every year in England**.

At what age do cataracts start to develop? (Out of **100** people)

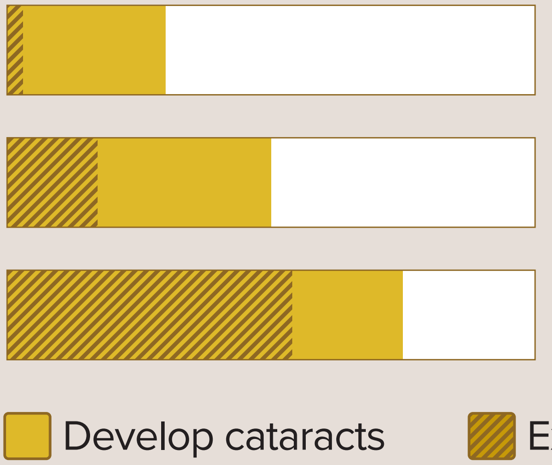

Age 30 – 40: Around **30** develop cataracts which cause symptoms in around **3**

Age 40 – 60: Around **50** develop cataracts which cause symptoms in around **17**

Age 60+: Around **75** develop cataracts which cause symptoms in around **54**

Symptoms

Over the years, **changes to the lens can cause symptoms**, such as:

- eye strain
- blurred or cloudy vision
- need brighter lights for reading
- double vision
- colours appear duller
- glare from bright lights
- difficulty driving / reading road signs

Cataracts usually affect both eyes, although they can develop faster in one eye than the other.

Some health problems can increase your risk of cataracts: past eye surgery or being very short-sighted, medical conditions like diabetes, or some medications, such as steroids.

Some cataracts are caused by sudden injury for example being hit directly in the eye. These cataracts tend to come on quite quickly (within weeks or months).

Have regular check ups at your optician. They can monitor progression of cataracts.

Regular eye examinations

Help detect cataracts and other eye problems at their earliest stages with regular eye examinations.

7 More information

What can I do to slow down the development of cataracts?

It may be many years from when you first notice changes in your vision, or an optometrist notices the start of cataracts, before they become a problem.

They may never worsen to the point where they affect your day-to-day life – the speed which cataracts develop varies from person to person.

Strong sunlight, cigarette smoke or drinking too much alcohol can all increase the chances of developing cataracts.

To help slow down development, or reduce the risk of cataracts becoming a problem

Stop smoking
Medications, counselling and other strategies are available to help you quit.

Manage other health problems
Follow your treatment plan if you have diabetes or other medical conditions that can increase your risk of cataracts.

Healthy diet
Choose a healthy diet that includes plenty of fruits and vegetables. A large study showed that a healthy diet rich in vitamins and minerals might help reduce the risk of developing cataracts.

Wear sunglasses
Ultraviolet light from the sun may contribute to the development of cataracts.

Reduce alcohol
Excessive alcohol use can increase the risk of cataracts.

8 More information

Vision aids and adaptations in more detail

There are many kinds of vision aids and adaptations which can help make the most of the vision that you have. If you choose to try them, advice, support and simple low vision aids are available from most opticians who can help you try different aids to see which work best for you.

Examples include

Plus lens magnifiers, that you can hold, wear as spectacles, or have on a stand. Some have built-in lights as well. These can help with reading or close work.

Real image magnifiers, which are cameras (CCTVs) that can show you an image on a screen that is magnified and enhanced. These are not available on the NHS, but many companies will let you try them out for free before buying them.

Visors, lenses and eye shields to reduce glare.

Changes in the home, such as changing lighting, colour schemes and putting white marks on the edges of steps or onto dials and switches.

Large-button devices such as phones.

Changing settings on computer and phone screens to improve contrast and size.

9 More information

Cataract surgery in more detail

- Usually you will be seen in an NHS hospital. Sometimes you might be seen in a private clinic offering NHS cataract operations.
- Before surgery, you'll be referred to a specialist eye team for an assessment. You will make a decision about the lens focus you want (either near or far). Talk to your specialist about whether you want to change the focus you currently have.

What will I feel during the surgery?

You will have to lie very still but you shouldn't feel any pain during surgery because your eye will be numbed first. You will not be able to see what is being done but you'll be able to hear the surgical team talking. Special fluid is used and you might feel this because it sometimes trickles down towards your ears.

What happens during the surgery?

Your eye is not removed to perform the surgery. The most common surgery is done using a small cut (incision) and a process called 'phacoemulsification' (phaco) which uses ultrasound to break up and remove your cloudy lens. The new plastic lens is put inside the eye. The cut in your eye is very small and most patients do not need stitches.

What happens after surgery?

You will not be able to drive yourself home and your vision will be blurry for up to 4 weeks. It might hurt a little. You might need some help at home because you won't be able to see clearly. Healing will usually take 4 – 6 weeks. You will have to use eye drops for this time and usually need a check up after the operation either at hospital or with your optometrist.

There are no major restrictions after surgery. Talk to your specialist about your own personal situation. You can do things like work, drive, exercise and fly as soon as you can see well enough.

Can I have both eyes done?

It may be possible to treat both eyes on the same day but it is more usual for surgery to be done several weeks apart to allow the recovery one eye at a time.

10 More information

Where can I go for more information?

Royal College of Ophthalmologists website has a useful section with questions and answers about cataracts that you might find helpful

www.rcophth.ac.uk

Royal National Institute for the Blind website gives you details on how to obtain their cataract information in audio format

www.rnib.org.uk

NHS

www.nhs.uk/conditions/cataract-surgery

Patient Info

https://patient.info/doctor/cataracts-and-cataract-surgery

Where did we get our numbers from?

- Benefits and risks of surgery from National Audit Data (NOD) which includes data about 600,000 surgeries (Clouding of the new lens data – page 19): https://www.nodaudit.org.uk/resources/publications-annual-report
- Benefits of vision aids from a review of studies by Cochrane: https://doi.org/10.1002/14651858.CD003303.pub4

Produced by:

Winton Centre for Risk and Evidence Communication and NHS England

Date last updated: July 2022 v1.1

Conflicts of Interest – None declared

Funding: NHS England

This decision aid was created with input from patients and healthcare professionals.

11 Contacts, further information, notes

Contacts

Who is my optometrist (optician)?	What are their contact details?
Who is my specialist at the hospital or clinic (ophthalmologist)?	What are their contact details?
What is the name of the clinic or hospital?	Contact details of hospital transport (if applicable)

Next steps

What will happen to me next? (treatments / tests?)

When will these happen?	When will I be reviewed next?

What decision do I need to make today?
Or when do I need to make a decision?

Questions for your specialist

These can be about any concerns you may have, for example: what you hope for from your treatment decision
